# Gastrointestinal comorbidities in patients with acne vulgaris: A population-based retrospective study

**DOI:** 10.1016/j.jdin.2024.08.022

**Published:** 2024-09-27

**Authors:** Yu-Wen Chen, Chun-Ying Wu, Yi-Ju Chen

**Affiliations:** a Department of Dermatology, Taichung Veterans General Hospital, Taichung, Taiwan; b Institute of Biomedical Informatics, Institute of Public Health, National Yang-Ming University, Taipei, Taiwan; c Faculty of Medicine and Institute of Clinical Medicine, National Yang-Ming University, Taipei, Taiwan; d Division of Translational Research and Center of Excellence for Cancer Research, Taipei Veterans General Hospital, Taipei, Taiwan; e Department of Public Health, China Medical University, Taichung, Taiwan; f National Institute of Cancer Research, National Health Research Institutes, Miaoli, Taiwan; g Department of Post-Baccalaureate Medicine, College of Medicine, National Chung Hsing University, Taichung, Taiwan

**Keywords:** acne vulgaris, antibiotics, constipation, gastroenteritis, gastroenterology, gastroesophageal reflux disease (GERD), gut-skin axis, irritable bowel syndrome (IBS), peptic ulcer

## Abstract

**Background:**

The gut-skin-brain axis has been long postulated in acne vulgaris. Few studies focused on bowel habits in patients with acne vulgaris have yielded controversial results.

**Objectives:**

To examine the relationship between acne vulgaris and gastrointestinal comorbidities.

**Methods:**

We conducted a nationwide case-control study using data from the Taiwan National Health Insurance Research Database spanning the years 1997 to 2013. Acne vulgaris and the control group were stratified by age, and we examined the association of gastrointestinal comorbidities across different age, sex, and antibiotic use through conditional logistic regression analysis.

**Results:**

A total of 185,491 patients with acne vulgaris were identified. The primary demographic for acne vulgaris comprised adolescents, followed by adult-onset groups, with a female predominance observed across all age subgroups. Patients with acne vulgaris exhibited a significantly elevated risk of developing gastrointestinal comorbidities, including peptic ulcers, irritable bowel syndrome, gastroenteritis, gastroesophageal reflux disease, and constipation. This increased risk was particularly notable in patients aged ≥12 years, and those with moderate-to-severe acne.

**Limitations:**

Miscoding and misclassification might have occurred.

**Conclusions:**

Patients with Acne vulgaris have higher risks of gastrointestinal comorbidities. For patients with moderate-to-severe acne, gastroenterology specialty consultation may be warranted.

**Capsule Summary**

• The gut-skin-brain axis has been long postulated in acne vulgaris.
• Patients with acne vulgaris have higher risks of gastrointestinal comorbidities. Gastrointestinal specialty consultation might be suggested in patients with moderate-to-severe acne for more comprehensive care.

## Introduction

Acne vulgaris affects approximately 9.4% of the global population.^1^ The pathogenesis of acne vulgaris is linked to abnormal follicular keratinization, microbial colonization with *Cutibacterium acnes*, inflammation, and increasing sebaceous glands secretion.^2^ Several comorbidities have been reported in association with acne vulgaris. Psychiatric conditions, particularly the relationship between depression, suicidal risk, and isotretinoin use, have been extensively discussed.^3^ Endocrine-associated diseases are implicated in adult-onset acne, predominantly affecting female populations.^4^

Although some studies have investigated on bowel habits in patients with acne vulgaris and irritable bowel syndrome (IBS), the results have been controversial results. Demirbaş and Elmas^5^ conducted a preliminary study of 300 patients with acne and 300 controls, revealing a significantly higher prevalence of IBS in individuals with acne vulgaris than healthy controls. However, in another case-control study, Daye et al^6^ examined the bowel habits of 100 patients with acne and matched controls, finding no correlation between irritable bowel diseases and acne and acne severity.

Little population-based research has focused on the relationship between acne vulgaris and gastrointestinal comorbidities. The objective of our study was to analyze the Taiwanese population, investigating the association between acne vulgaris and gastrointestinal comorbidities.

## Materials and methods

### Data source

The study was conducted based on the information released from Taiwan’s National Health Insurance Research Database (NHIRD). This extensive database, providing coverage for 99% of the Taiwanese population, encompasses comprehensive records of both inpatient and outpatient visits, offering detailed documentation on laboratory tests, medications, and procedures. Personal information, including body weight, height, family history, lifestyle and habits, or laboratory results are not available from the database. Diagnoses were coded using the International Classification of Diseases, ninth revision (ICD-9) code during the specific study period.

### Study population

The data in this study were derived from the Longitudinal Health Insurance Database 2000 and 2010, both subsets encompassing 1 million patients sourced from the NHIRD. The study subjects were identified based on the diagnosis of acne vulgaris (ICD-9-clinical modification [CM] code 706.1) from 1997 to 2013 in the outpatient service. The index date was defined as the initial occurrence of an acne vulgaris diagnosis. To ensure the accuracy of acne vulgaris diagnosis, only patients with a minimum of 3 outpatient visits were included. For control group, individuals without a diagnosis of acne vulgaris throughout the study period were randomly selected. Matching was performed for age and sex to assign an index date for the control group, ensuring a comparable starting point for both case and control groups. Additionally, we excluded potential acne mimickers,^7^ including rosacea (ICD-9-CM code 695.3), acneiform drug eruption (ICD-9-CM code 692.3), and cutaneous lupus erythematosus (ICD-9-CM code 695.4). Patients with cancer were also excluded from the study population.

**Fig 1:**
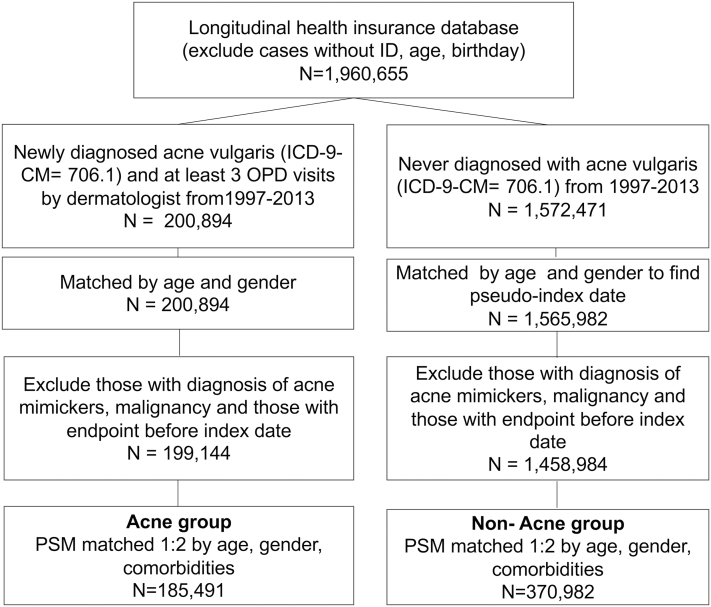
The flowchart of the study design.

The study group comprised of 185,491 patients with acne vulgaris, with 370,982 age- and sex-matched individuals (Fig 1). The end point for all study subjects was either December 31, 2013 or the occurrence of a death event, whichever transpired first. The selected population was further stratified into 3 groups based on age: childhood (<12 years old), adolescence (12-25 years old), and adulthood (>25 years old). Oral antibiotics use was defined as extended use of minocycline, tetracycline, and doxycycline for a duration exceeding 30 days. Patients with oral antibiotic use exceeding 30 days were classified as moderate-to-severe cases. Notably, systemic isotretinoin was not included in our analysis because of its strict regulations regarding reimbursement in the National Health Insurance. Most of the systemic isotretinoin usage in Taiwan is self-paid, and such information may not be discernible in the NHIRD.

### Gastrointestinal comorbidities associated with acne vulgaris

For inclusion in the study as individuals with specific disease, patient was required a minimum of 3 diagnoses made by a physician specializing in the relevant field. Specifically, the newly diagnosed gastrointestinal diseases, namely peptic ulcers (ICD-9-CM code 533.9), IBS (ICD-9-CM code 564.1), gastroenteritis (ICD-9-CM code 558.9), gastroesophageal reflux disease (GERD) (ICD-9-CM code 530.81), and constipation (ICD-9-CM code 564.0) were diagnosed by board-certificated specialists, including gastroenterologists and colon-rectal surgeons.

### Statistical analysis

The demographic data of the study population were analyzed. Continuous data were presented as median with IQR. For between-group comparisons, the Student *t* test was employed for normally distributed continuous variables, whereas the Wilcoxon rank sum test was used for continuous variables that did not adhere to normal distribution. The Pearson χ^2^test or Fisher’s exact test was applied for the analysis of nominal data as appropriate.

The associated gastrointestinal comorbidities of the overall condition, both before the index date and before the end date, were calculated. To access the magnitude of the associations between individual diseases and acne vulgaris, logistic regressions were conducted to determine the odds ratio and 95% CI. A 2-tailed *P* value of <.05 was considered statistically significant. The statistics analysis package (SAS for Windows, version 9.4, SAS Institute Inc) was used for data analysis.

## Results

A total of 199,144 subjects were diagnosed with acne vulgaris from 2000 to 2013. Among these, 8786 (4.4%) were diagnosed at the age of <12 years, 127,096 (63.8%) were diagnosed between the ages of 12 and 25 years, and 63,262 (31.8%) were diagnosed at the age of >25 years. The matched control patients totaled 1,458,984. The predominant demographic for acne vulgaris was adolescents, followed by adult-onset groups, with a female predominance in all categories. Within the acne group, 37.3% of the patients had received treatment with systemic antibiotics. Notably, adolescents exhibited a higher percentage (41%) individuals with extended antibiotics usage, which indicated moderate-to-severe acne (Table I).

**Table I:** Demographic data of the study population

Characteristics	Mild acne subgroup						Moderate-to-severe acne subgroup					
	<12 y/o		12-25 y/o		≥25 y/o		<12 y/o		12-25 y/o		≥25 y/o	
	Case	Control	Case	Control	Case	Control	Case	Control	Case	Control	Case	Control
	*N* = 6499	*N* = 12,998	*N* = 68,256	*N* = 136,512	*N* = 41,554	*N* = 83,108	*N* = 2287	*N* = 4574	*N* = 47,653	*N* = 95,306	*N* = 19,242	*N* = 38,484
Age, y, mean ± SD	10.3 ± 1.9	10.3 ± 1.9	18.0 ± 3.6	18.0 ± 3.6	36.7 ± 10.2	36.7 ± 10.2	10.8 ± 1.4	10.8 ± 1.4	18.2 ± 3.4	18.2 ± 3.4	34.7 ± 9.1	34.7 ± 9.1
Follow-up, y, mean ± SD	7.6 ± 3.6	3.8 ± 3.4	8.2 ± 4.1	8.5 ± 4.4	8.4 ± 4.0	10.2 ± 3.8	9.8 ± 3.2	3.9 ± 3.5	10.5 ± 3.5	8.5 ± 4.4	10.5 ± 3.5	10.4 ± 3.8
Female	4536 (69.8%)	9072 (69.8%)	38,879 (57.0%)	77,758 (57.0%)	30,031 (72.3%)	60,062 (72.3%)	1548 (67.7%)	3096 (67.7%)	26,084 (54.7%)	52,168 (54.7%)	13,489 (70.1%)	26,978 (70.1%)
Male	1963 (30.2%)	3926 (30.2%)	29,377 (43.0%)	58,754 (43.0%)	11,523 (27.7%)	23,046 (27.7%)	739 (32.3%)	1478 (32.3%)	21,569 (45.3%)	43,138 (45.3%)	5753 (29.9%)	11,506 (29.9%)
Oral form antibiotic(≥30 d)	NA	41 (0.3%)	NA	2170 (1.6%)	NA	3,038 (3.7%)	2287 (100.0%)	22 (0.5%)	47,653 (100.0%)	1465 (1.5%)	19,242 (100.0%)	1436 (3.7%)

*NA*, Not applicable; *y/o*, years old.

### Gastrointestinal comorbidities

**Fig 2:**
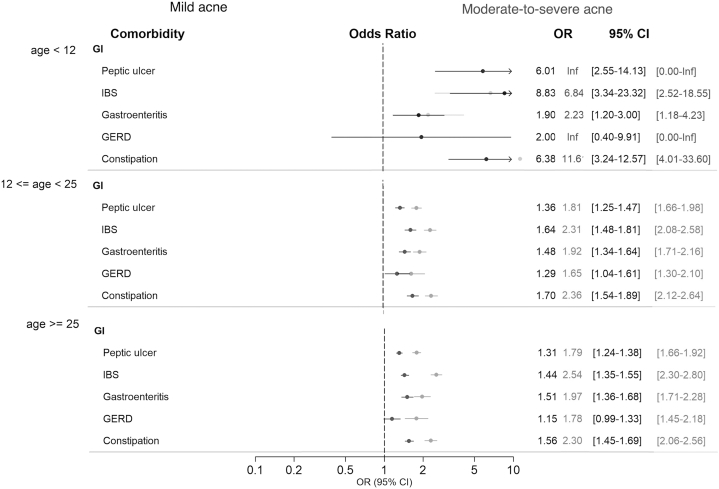
The overall gastrointestinal comorbidities in patients with acne vulgaris. Mild acne group defined as patients with acne vulgaris who did not use antibiotics. Moderate-to-severe acne group was defined as patients with acne vulgaris who used antibiotics (minocycline, doxycycline, and tetracycline) for >30 days. In patient aged >12 years, peptic ulcer, irritable bowel syndrome (IBS), gastroenteritis, gastroesophageal reflux disease, and constipation are bidirectionally associated with acne vulgaris. Higher odd ratio in the subgroup of more severe disease activity and with antibiotics use. As for children, IBS and constipation are most related with acne vulgaris.

The comprehensive overview of gastrointestinal comorbidities was presented in Fig 2. Patients with Acne cross all age groups exhibited a propensity for increased prevalence of gastrointestinal comorbidities, including peptic ulcers, IBS, gastroenteritis, GERD, and constipation. Among patients aged >12 years, those with systemic antibiotics usage demonstrated a more pronounced association with gastrointestinal comorbidities compared with the nonantibiotics group (Fig 2, Supplementary Table I, available via Mendeley at https://data.mendeley.com/datasets/fsxf7mx8y4/1).

Additionally, patients with acne vulgaris tended to have a pre-existing history of gastrointestinal diseases. In individuals aged >12 years, irrespective of antibiotic exposure, a correlation was observed between gastroenteritis, IBS, GERD, constipation, and peptic ulcers. Among children aged <12 years, IBS and constipation were related to the development of acne vulgaris, albeit without statistical significance (Supplementary Fig 1, Supplementary Table II, available via Mendeley at https://data.mendeley.com/datasets/fsxf7mx8y4/1). The analysis presented in Figure 2, Supplementary Table III (available via Mendeley at https://data.mendeley.com/datasets/fsxf7mx8y4/1) revealed that patients with acne exhibited a heightened risk of future gastrointestinal conditions compared with controls. Among patients aged >12 years, those with a more severe manifestation of acne, as indicated by using antibiotics usage, demonstrated a higher risk of developing peptic ulcers, IBS, gastroenteritis, GERD, and constipation. The heightened risk was more pronounced in individuals aged <12 years.

## Discussion

Our 13-year population-based study revealed that patients with acne have an overall elevated risk of experiencing gastrointestinal comorbidities, including peptic ulcers, IBS, constipation, gastroenteritis, and GERD, regardless of age difference. There was a robust association between gastrointestinal conditions and acne vulgaris, especially IBS in those aged <12 years. Moreover, the association of gastrointestinal comorbidities was greater in adolescents and young adults with moderate-to-severe acne.

Stokes and Pillsbury^8^ first presented the “gut-brain-skin” theory in 1930. The authors hypothesized a connection between emotional status, neurotransmitters, alternation in intestinal permeability, changes in microbial flora, and disruptions in the balance between the skin and gut. These interrelated factors would contribute to local or systemic inflammation, consequently leading to various cutaneous diseases, including urticaria, atopic dermatitis,^9^ psoriasis,^10^ and acne vulgaris. Other pilosebaceous unit diseases were also associated with gastrointestinal comorbidities. Specifically, hidradenitis suppurativa was associated with individuals with IBS,^11^ whereas rosacea was observed to be related to inflammatory bowel syndromes, *Helicobacter pylori* infection, IBS, and celiac disease.^12^

Gastrointestinal comorbidities observed in individuals with acne vulgaris may manifest either preceding or after the onset of acne. The alternation of gut microbiome is suggested to play a role in acne vulgaris development and severity. Hypochlorhydria, observed in patients with acne, has been linked to the migration of colonic bacteria to the small intestine, resulting in abnormal bacterial overgrowth that affects food digestion and contributes to systemic oxidative stress.^13^ The composition of patients with acne vulgaris differs from health controls, exhibiting decreased diversity with a lower abundance of *Firmicutes* and increased *Bacteroides*.^14^ Upregulation of lipopolysaccharides biosynthesis pathway has been observed in the acne-affected population, potentially attributed to an increase in lipopolysaccharides-producing bacteroides.^15^

Saleh et al^16^ reported that individuals with severe acne vulgaris not only have a higher incidence of *H pylori* infection but also exhibit elevated levels of *H pylori* antigen in fecal material and *H pylori* antibodies in serum than those with mild-to-moderate acne. These findings are consistent with our study, providing additional support to the association between peptic ulcers and acne vulgaris.

Patients treated with antibiotics exhibited a higher likelihood of having gastrointestinal comorbidities than those who did not use antibiotics. This trend may be attributed to disease severity or the impact of systemic antibiotics, which disrupted the balance of the microbiome in both the skin and gut. Thompson et al^17^ discovered that oral minocycline caused disarrangement of skin and gut microbiome. The depletion of multiple probiotic species, such as *Lactobacillus salivarius*, *Bifidobacterium adolescentis*, *Bifidobacterium pseudolongum*, and *Bifidobacterium breve*, further contributed to inflammatory acne formation, as these species played natural roles in reducing gut inflammation. Additionally, the depletion of *Staphylococcus epidermidis*, a biofilm-producing gram-positive coccus inhibiting *C acnes* growth, could induce inflammation on the skin. Certain strains of *C acnes* were found to determine the onset of acne vulgaris rather than the quantity of bacteria.^18^ Therefore, the dosage, duration, and action mechanism of oral antibiotics should be considered carefully before prescribing them to patients with acne, as prolonged and nonselective antibiotics use disturbs the balance of skin and gut microbiome as well as promotes the growth of antibiotics-resistant species, including *C acnes*.^19^

Our research deprived data from NHIRD, in which acne vulgaris severity grading and isotretinoin prescription were not documented. Global Acne Grading System and Investigator Global Assessment of Acne are among the most frequently used grading systems to assess acne severity.^20^ However, some research relies on self-reported severity. Clinical advice suggesting consecutive use of tetracyclines as treatment in our research might indicate a more severe condition in patients with acne.^21^ Whether antibiotics exposure is related to disease activity would require another study design for clarification.

The influence of diet on acne has long been postulated. Our result revealed a significant association between gastrointestinal comorbidities and acne vulgaris, particularly in patients aged <12 years, with IBS and constipation showing notable associations. Our previous study in Taiwan indicated an elevated prevalence of acne among individuals aged <12 years,^22^ potentially attributed to the increasing popularity of a Western diet with a relatively high glycemic index.^23^ The consumption of such diet has been linked to the production of insulin and insulin-like growth factors, which, in turn, induce acne formation by promoting the proliferation of sebocytes and keratinocytes and enhancing lipid synthesis.^24^

This research provides an early population-based exploration of the association between acne vulgaris and gastrointestinal comorbidities, providing support for the “gut-skin axis theory” in the pathogenesis of these diseases. Despite the value insights gained, several limitations should be acknowledged. Clinical information, including the family history, lesion location, acne severity aside from oral antibiotics use, body mass index, diet habits, lifestyle, and self-paid treatment such as isotretinoin use, could not be retrieved from NHIRD. The potential for misclassification of diseases on NHIRD could not be completely excluded. To mitigate the selection bias, we ensured a robust selection process, relying on multiple diagnoses by specialists for both acne and gastrointestinal comorbidities. Lastly, the findings were based on the Taiwanese population and may not be generalized to other races and populations.

In conclusion, our present study demonstrated that gastrointestinal comorbidities were more common in patients with acne than nonacne controls, with a stronger association observed among those aged <12 years and those with moderate-to-severe acne.

## Conflicts of interest

None disclosed.
